# Deubiquitinating Enzymes as Therapeutic Candidates in Hepatocellular Carcinoma and Other Liver Disease

**DOI:** 10.3390/ijms27125625

**Published:** 2026-06-22

**Authors:** Young-Hoon Jeong, Hwa-Hyeong Lee, Young-Jun Kim, Hye-Rim Lee, Key-Hwan Lim

**Affiliations:** College of Pharmacy, Chungbuk National University, Osongsaengmyeong 1-ro, Osong-eup, Heungdeok-gu, Cheongju-si 28160, Republic of Korea; steve8891@naver.com (Y.-H.J.);

**Keywords:** hepatocellular carcinoma, deubiquitinating enzymes, ubiquitin–proteasome-system, protein homeostasis, therapeutic targets

## Abstract

Hepatocellular carcinoma is challenging to detect at an early stage, and its severity increases over time. Recently, the incidence of hepatocellular carcinoma has increased, partly due to lifestyle-related factors such as excessive alcohol intake, sedentary behavior, and diets high in fat, which contribute to the growing prevalence of fatty liver and hepatitis. Various therapeutic strategies are being explored for hepatocellular carcinoma, among which therapies targeting deubiquitinating enzymes (DUBs) have attracted growing attention. Ubiquitination acts as a crucial modulator in the regulation of intracellular signaling across many diseases. E3 ligase recognizes the target protein and transfers ubiquitin, received from the E2 enzyme, to the lysine residues of the substrate, thereby conferring specificity to the ubiquitination process. Once a ubiquitin chain is attached to a target protein by an E3 ligase, the protein is directed to the ubiquitin–proteasome system (UPS) for degradation. In this process, the 26S proteasome complex recognizes the ubiquitin chain and degrades the target protein, thereby serving as a major mechanism for maintaining protein homeostasis. Through this pathway, cells regulate signal transduction, eliminate abnormal proteins, and perform various essential functions. On the other hand, deubiquitinating enzymes (DUBs) recognize the ubiquitin chains on target proteins and remove them by hydrolyzing the isopeptide bonds of ubiquitin, thereby enabling the target proteins to evade degradation by the proteasome system. Furthermore, deubiquitinating enzymes independently remove ubiquitin from proteins and can serve as central regulators in signaling pathways related to hepatocellular carcinoma.

## 1. Introduction

**Figure 1:**
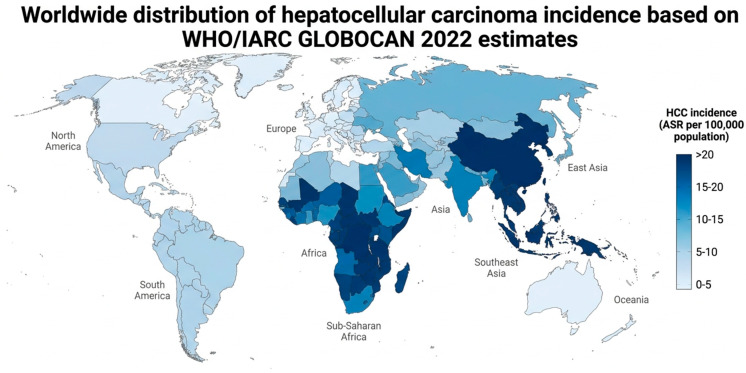
Worldwide distribution of hepatocellular carcinoma incidence based on WHO/IARC GLOBOCAN 2022 estimates. Global distribution of hepatocellular carcinoma (HCC) incidence based on age-standardized incidence rates (ASRs) per 100,000 population derived from the WHO/IARC GLOBOCAN 2022 database. Higher incidence rates were predominantly observed in East Asia, Southeast Asia, and sub-Saharan Africa, reflecting regional differences in the prevalence of chronic hepatitis B virus (HBV) and hepatitis C virus (HCV) infections, alcohol-related liver disease, and non-alcoholic fatty liver disease (NAFLD). Abbreviations: HCC, hepatocellular carcinoma; ASR, age-standardized rate [3].

Liver cancer ranks among the most fatal cancers globally, contributing to approximately 900,000 new diagnoses and 830,000 deaths each year around the world [1]. Liver cancer is the fifth most common cancer in men and ranks third in global cancer-related mortality. It also shows a high recurrence rate of over 70% within five years after treatment [2]. There are various types of liver cancer, but they can largely be classified into two main forms. Hepatocellular carcinoma accounts for about 75% of all cases, while intrahepatic cholangiocarcinoma represents around 15%. Liver cancer commonly develops in individuals with pre-existing liver conditions such as fatty liver or cirrhosis, and its risk factors vary depending on the underlying cause and geographic region. According to the 2022 estimates published by the World Health Organization (WHO), hepatocellular carcinoma (HCC) showed particularly high incidence rates in East Asia, Southeast Asia, and sub-Saharan Africa (Figure 1) [3].

**Figure 2:**
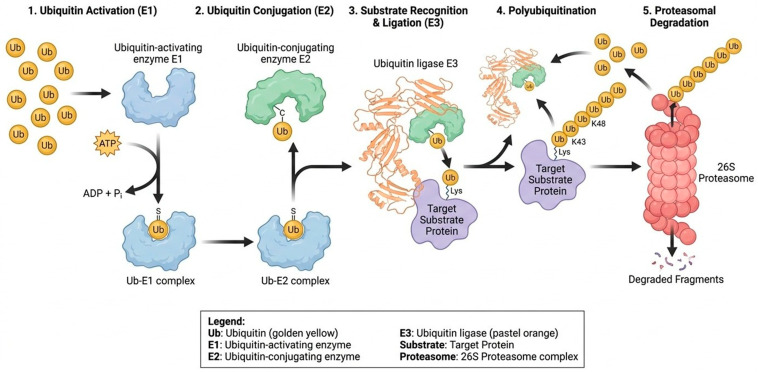
Mechanisms of ubiquitination and deubiquitination. This figure illustrates the process of ubiquitination, in which ubiquitin chains are conjugated to a substrate protein. Ubiquitin chains are attached by the enzymatic cascade of E1, E2, and E3 ligases, after which the 26S proteasome mediates degradation of the substrate protein. However, when deubiquitinating enzymes (DUBs) intervene, the ubiquitin chains are removed or destabilized, preventing proteasomal recognition. As a result, the substrate protein evades degradation and becomes stabilized.

Studies show that hepatocellular carcinoma incidence is rising in the United States and Central Europe. This may be due to the obesity epidemic caused by modern dietary habits and lack of exercise, as well as the continued spread of hepatitis. In this context, alcohol-related cirrhosis and non-alcoholic fatty liver disease are considered as potential precursors to hepatocellular carcinoma [2]. The progression from hepatitis to cirrhosis and ultimately to hepatocellular carcinoma represents a major and commonly observed pathway in hepatic disease. Chronic liver injury, often triggered by hepatitis or other pathological factors, promotes the activation of hepatic stellate cells and the accumulation of extracellular matrix components, which can eventually lead to the development of hepatocellular carcinoma [1]. For instance, non-alcoholic steatohepatitis is known to involve not only fat accumulation in the liver but also inflammatory responses. Additionally, long-term follow-up studies have indicated that approximately 5% of individuals with fatty liver disease may eventually develop cirrhosis or hepatocellular carcinoma [4]. Therapeutic approaches for treating hepatocellular carcinoma include systemic therapy with multikinase inhibitors and immune checkpoint inhibitors. The use of multikinase inhibitors in systemic treatment provides limited survival advantage, which is often negated by notable adverse effects. Immune checkpoint blockade represents a promising new approach for HCC and can enhance patient survival when used with targeted agents, though its response rate remains below 50% [5]. In this situation, therapies targeting deubiquitinating enzymes are currently receiving growing attention. Ubiquitination refers to the process of attaching ubiquitin molecules to a target protein [6]. In cells, protein ubiquitination proceeds through a sequential reaction involving E1 (ubiquitin-activating enzyme), E2 (ubiquitin-conjugating enzyme), and E3 (ubiquitin ligase). Within this process, E1 utilizes ATP to form a thioester bond between its catalytic cysteine and the C-terminal of ubiquitin. Subsequently, the ubiquitin is relayed to the E2 enzyme, and E3 facilitates the transfer of ubiquitin to the designated substrate by interacting with E2 (Figure 2) [7].

The interplay between protein tagging and removal of ubiquitin within the ubiquitin–proteasome system (UPS) serves as a crucial post-translational mechanism regulating the stability and degradation of proteins involved in epithelial–mesenchymal transition [8]. This occurs when the carboxyl terminus of ubiquitin forms a covalent bond with the amine group of a lysine residue on the substrate. Additionally, ubiquitin can generate polymeric chains through one of its seven intrinsic lysine residues or its initial methionine (M1) [6]. The topology of polyubiquitin chains varies based on which ubiquitin residue is involved, influencing the downstream fate of the tagged protein. For instance, chains formed through lysine residues at position K48 typically signal the substrate for breakdown via the proteasome. In contrast, chains linked through lysine residues at position K63 direct the removal of damaged cellular components through the autophagy–lysosome system [9]. According to previous studies, the ubiquitin–proteasome system is a major mechanism of protein degradation. It also serves as a key regulator of pathways involved in DNA repair, cell proliferation, and responses to various forms of stress [7]. The process of deubiquitination is driven by deubiquitinating enzymes (DUBs), which remove ubiquitin from target proteins, ultimately hindering the function of the ubiquitin–proteasome system (Figure 2) [10]. Although roughly 90 deubiquitinating enzymes (DUBs) have been discovered, the recent emergence of atypical DUB families implies the existence of additional ones [11]. The types of deubiquitinating enzymes include UCH (ubiquitin carboxy-terminal hydrolase), USP (ubiquitin-specific protease), OTU (ovarian tumor protease), and the Josephin domain; in contrast, the JAMM (MPN+) class is categorized under the zinc-dependent metalloprotease family [12]. Despite their limited number, these enzymes play a central role in fine-tuning ubiquitinated proteins and ultimately serve as key regulators of cellular activities, organismal development, and disease-related mechanisms [11]. Accumulating evidence has demonstrated that deubiquitinating enzymes (DUBs) contribute to the pathogenesis of numerous diseases. In particular, dysregulated expression and activity of DUBs have been frequently observed in immune disorders and malignancies, highlighting their potential as promising therapeutic targets [13]. Notably, numerous DUBs have been reported to exhibit aberrant expression in HCC, where they contribute to tumor progression by regulating cellular proliferation and facilitating resistance to currently available therapeutic agents. Moreover, accumulating evidence indicates that certain DUBs, including USP14, play important roles in shaping the hypoxic tumor microenvironment, thereby influencing HCC development and therapeutic responses [14]. For the successful clinical translation of DUB-targeted therapies, the development of highly selective inhibitors against specific DUBs is essential [15]. Several obstacles hinder the clinical translation of DUB-targeted therapies. The structural conservation of catalytic domains among DUB family members limits the development of highly selective inhibitors. Furthermore, because individual DUBs regulate multiple cellular signaling pathways, off-target effects remain a major concern. Consequently, comprehensive evaluations of both therapeutic efficacy and cytotoxicity are required to ensure the safety of DUB-based therapeutic strategies [16]. This paper aims to provide an overview of studies in which deubiquitinating enzymes act as key regulatory factors in the pathogenesis of hepatocellular carcinoma and liver diseases.

## 2. DUB Regulators of Liver Diseases

Hepatocellular carcinoma (HCC) is one of the most common malignancies worldwide and is associated with a very high mortality rate. Early diagnosis of HCC remains challenging, and due to its rapid progression, the prognosis is generally poor with low survival rates. Although hepatic resection and liver transplantation are currently the main therapeutic options, these approaches are not suitable for some patients due to their substantial physiological burden [14]. Thus, DUBs are increasingly recognized as pivotal drivers of HCC, alongside the clinical advancement of several DUB-targeting inhibitors [17].

### 2.1. USP1

The USP1 gene has been implicated in DNA damage repair by promoting the assembly of DNA replication forks [18]. Elevated USP1 expression has been observed across multiple malignant tumors, while its suppression has been demonstrated to inhibit tumor growth in HCC and various other cancer types. Additionally, inhibition of USP1 activity has been shown to promote G0/G1 phase cell cycle arrest, induce apoptosis, and trigger autophagy. It was further confirmed that blocking USP1 can suppress metastatic behavior in hepatocellular carcinoma (HCC). Previous findings have also revealed that USP1 stabilizes SIX1 by removing ubiquitin, thereby modulating cyclin D1 expression (Table 1). Pharmacological or genetic inhibition of USP1 using agents such as ML-323 or siRNA led to reduced levels of both SIX1 and cyclin D1 proteins. SIX1 functions as a transcription factor that regulates cancer cell proliferation and metastasis, while cyclin D1 is a key cell cycle regulator that facilitates the transition from G1 to S phase [19]. Ultimately, elevated expression of USP1 allows both SIX1 and cyclin D1 to evade degradation by the ubiquitin–proteasome system (UPS), thereby promoting tumor cell proliferation and accelerating cell cycle progression [20]. In addition, several USP1 inhibitors, including KSQ-4279, SIM0501, and XL309-101, have been developed and are currently undergoing preclinical or early clinical evaluation. Preclinical studies have demonstrated that USP1 inhibition can restore sensitivity to platinum-based chemotherapy in tumors that have acquired platinum resistance. Among these agents, KSQ-4279 has shown promising pharmacokinetic and pharmacodynamic properties, along with encouraging preliminary clinical activity. Early-phase clinical studies have also suggested an acceptable safety profile, with anemia reported as the most frequently observed adverse event [21].

### 2.2. USP7

USP7 is frequently upregulated in several human malignancies, including lung cancer, prostate cancer, and hepatocellular carcinoma. Unlike many DUBs that regulate a limited number of substrates, USP7 modulates both the tumor suppressor p53 and its negative regulator MDM2 through deubiquitination. By controlling the stability of these key proteins, USP7 exerts broad effects on cell cycle regulation, apoptosis, and tumor progression in multiple cancer types [22]. USP7 is frequently overexpressed in hepatocellular carcinoma (HCC), suggesting its potential as a therapeutic target. Elevated USP7 expression has been supported by transcriptomic datasets and immunohistochemical analyses and has been associated with immune-related pathways, including JAK/STAT and VEGF signaling [23]. USP7 was highly expressed in HCC tissues and cell lines and showed a positive association with TRIM27 expression and STAT3 activation. Co-immunoprecipitation experiments confirmed the formation of a JAK1–TRIM27–USP7 complex. Furthermore, depletion or inhibition of USP7 reduced TRIM27 levels and attenuated STAT3 signaling, suggesting that USP7 contributes to HCC progression through the TRIM27/STAT3 axis [24]. The USP7 inhibitors investigated to date, OAT-4828 has demonstrated the ability to enhance antitumor immunity by reducing the expression of immunosuppressive molecules, including PD-L1. These findings suggest that USP7 inhibition may improve the responsiveness to immune checkpoint inhibitor (ICI)-based therapies and potentially overcome resistance mechanisms that limit their clinical efficacy [25]. Previous studies have also demonstrated that USP7 promotes HCC cell survival and invasion. Therapeutically, inhibition of USP7 enhances tumor cell apoptosis and has been linked to pathways involving BRAF, MEK, and PARP. However, clinical evidence regarding treatment efficacy and safety remains limited. In addition, the broad substrate spectrum and complex regulatory functions of USP7 make it difficult to define its specific contributions within interconnected signaling networks, highlighting the need for further mechanistic and translational studies [26].

#### USP7-Mediated Regulation of NF-κB/STAT3 Signaling in HCC

Hepatocellular carcinoma (HCC) remains a leading cause of cancer-related mortality worldwide because of its high metastatic potential and limited therapeutic responsiveness. Among the key mechanisms driving HCC progression, the NF-κB/STAT3 signaling axis promotes tumor proliferation, invasion, and metastasis. Persistent NF-κB activation induces IL-6 production, leading to STAT3 activation and the establishment of a positive feedback loop that sustains chronic inflammation and hepatocarcinogenesis [27]. Emerging evidence suggests that dysregulation of deubiquitinating enzymes (DUBs) contributes to aberrant NF-κB/STAT3 signaling, highlighting this pathway as a promising therapeutic target in HCC [28]. In addition, AKR1C3 has been shown to facilitate TRAF6 ubiquitination, thereby stimulating NF-κB activity and promoting the secretion of inflammatory mediators. These cytokine-driven signals subsequently enhance STAT3 phosphorylation, ultimately accelerating HCC cell growth and invasive behavior [29]. As STAT3, a central mediator of this oncogenic feedback loop, is regulated by upstream TRIM27-dependent signaling and associated with enhanced p-JAK1 activation, USP7-mediated stabilization of TRIM27 may contribute to HCC progression. Therefore, USP7 has potential utility as both a prognostic biomarker and a therapeutic target in HCC [24].

### 2.3. USP11

A comprehensive screening of human deubiquitinating enzymes identified USP11 as a key driver in tumor initiation and development. USP11 has been shown to enhance the proliferation and metastatic potential of colorectal and hepatocellular carcinoma by preserving NF90 stability and stimulating the ERK/MAPK pathway (Table 1) [5]. Previous studies have shown that USP11 can function either as a tumor suppressor or an oncogene depending on the tissue context. For instance, in colorectal, breast, and hepatocellular carcinoma, USP11 expression has been associated with unfavorable clinical outcomes. Conversely, in glioma, USP11 suppresses tumor growth and invasiveness [30]. Previous studies uncovered that the USP11–eEF1A1–SP1–HGF–AKT axis plays a significant role in enhancing EMT and metastatic behavior in hepatocellular carcinoma [5]. Eef1a1 serves crucial functions in the translation process by delivering aminoacyl-tRNA to the ribosome’s A-site. Beyond this fundamental role, eEF1A1 and eEF1A2 are also involved in diverse cellular processes such as cytoskeletal organization, regulation of cell growth and death, and protein degradation via proteasome pathways. Given these versatile functions, both isoforms have been implicated in oncogenesis, where they are considered markers of cellular transformation and tumor advancement, including in hepatocellular carcinoma as potential prognostic indicators [31]. USP11 was found to associate with eEF1A1 and remove its polyubiquitin chains at lysine 439, leading to elevated eEF1A1 levels. This upregulated eEF1A1 then bound to SP1, promoting the transcription of HGF and consequently stimulating the AKT/Snail cascade to drive EMT and tumor dissemination [5]. Although no selective USP11 inhibitors have been identified to date, mitoxantrone, an established anticancer drug, has been reported to inhibit USP11 through off-target mechanisms [32]. In preclinical colorectal cancer models with elevated USP11 expression, mitoxantrone treatment suppressed EGFR- and TLR-mediated signaling pathways and attenuated tumor progression in tumor-bearing mice [33].

### 2.4. USP14

**Figure 3:**
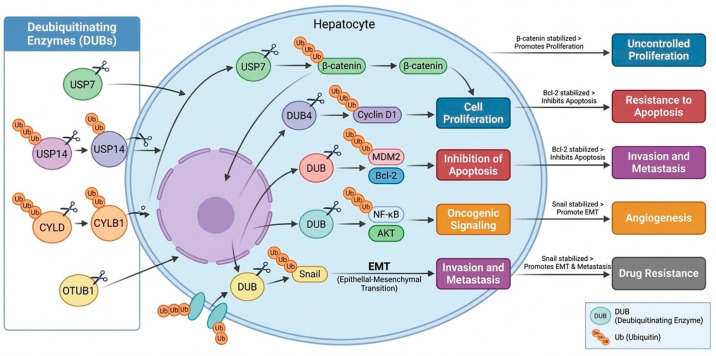
Schematic illustration of DUB-mediated regulation of hepatocellular carcinoma hallmarks. DUBs promote HCC progression by deubiquitinating and stabilizing oncogenic substrates, including β-catenin, Cyclin D1, MDM2, Bcl-2, NF-κB, AKT, and Snail. These events contribute to enhanced cell proliferation, inhibition of apoptosis, activation of oncogenic signaling, epithelial–mesenchymal transition (EMT), invasion and metastasis, angiogenesis, and drug resistance, ultimately driving HCC progression. Ub indicates ubiquitin.

USP14 has emerged as an important deubiquitinating enzyme (DUB) implicated in a variety of pathological conditions, including neurodegenerative disorders and malignancies. Through the stabilization of key substrate proteins, USP14 contributes to the regulation of multiple cellular processes and is frequently found to be aberrantly expressed in diverse disease settings [34]. Recent studies have identified USP14 as a critical regulator of HCC progression, with evidence demonstrating its involvement in tumor cell proliferation, migration, and invasion. Mechanistically, USP14 has been shown to modulate both AKT and epithelial–mesenchymal transition (EMT) signaling pathways at the protein level. Furthermore, genetic silencing or pharmacological inhibition of USP14 resulted in suppression of these oncogenic pathways. Given the established roles of AKT and EMT signaling in tumor initiation (Figure 3), metastatic dissemination, and therapeutic resistance, the USP14-mediated regulatory axis may represent an important contributor to HCC progression and a potential target for therapeutic intervention [35]. Current therapeutic strategies for HCC include surgical resection, local ablation, radiotherapy, and systemic drug treatment. However, the effectiveness of radiotherapy is often limited by the intrinsic heterogeneity and radioresistance of HCC cells. Recent studies have identified USP14 as a critical regulator of radioresistance through its involvement in ferroptosis-associated signaling pathways. Mechanistically, USP14 contributes to the maintenance of the SLC7A11–GSH–GPX4 ferroptosis defense axis, thereby promoting tumor cell survival under radiation-induced oxidative stress. In particular, TRIM14 recruits USP14 to GPX4, enabling USP14-mediated deubiquitination of K48-linked ubiquitin chains on GPX4. This process prevents proteasomal degradation of GPX4, preserves its anti-ferroptotic activity, and ultimately enhances the resistance of HCC cells to radiotherapy [36]. Early efforts to target USP14 led to the identification of b-AP15, a compound capable of inhibiting both USP14 and UCHL5. However, its therapeutic application was limited by insufficient selectivity toward USP14. To address this issue, the more potent analog VLX1570 was subsequently developed, although its clinical development was hindered by reports of significant toxicity. Later, the selective USP14 inhibitor IU1 was introduced and demonstrated substantially improved target specificity. Further optimization of this scaffold resulted in the development of next-generation compounds, including IU1-47, which exhibited enhanced inhibitory potency and greater selectivity for USP14 [37].

### 2.5. USP19

Elevated expression of USP19 and SOAT1 is observed in human HCC and is associated with an unfavorable prognosis. ChIP–qPCR and luciferase reporter assays revealed that USP19 and SOAT1 are transcriptionally regulated by P53. Mechanistically, USP19 removes ubiquitin from SOAT1 at K33 and K48 residues, enhancing its protein stability (Table 1) [38]. SOAT1 is one of the isoforms of Sterol O-acyltransferases, resides in the endoplasmic reticulum and functions in dimeric or tetrameric form to convert cholesterol into its esterified form. Inhibition of SOAT1 has been shown to hinder the progression of several cancers, including glioblastoma, pancreatic, colorectal, and gastric cancers [39]. From a functional standpoint, inhibiting SOAT1—but not USP19—diminishes tumor formation in P53-deficient HCC. Collectively, these findings indicate that the P53–USP19–SOAT1 axis governs cholesterol esterification involved in liver tumor progression. Targeting SOAT1 may serve as an effective therapeutic approach in P53-deficient liver malignancies [38]. Collectively, these findings identify the p53–USP19–SOAT1 axis as a potential therapeutic vulnerability in p53-deficient HCC and warrant further investigation into strategies targeting this pathway.

### 2.6. USP22

According to previous studies, elevated USP22 in HCC prevents degradation of PPARγ, leading to its accumulation, which activates Akt2 and promotes both lipogenesis and tumorigenesis (Table 1) [40]. In the early 1990s, first described PPARγ, a nuclear hormone receptor encoded on human chromosome 3p25.2, now known to be a key modulator of adipose tissue biology. This gene is composed of nine exons and undergoes alternative splicing and promoter selection to produce four transcript variants, which result in two main protein isoforms. PPARγ2, which includes 28 additional residues at the N-terminal end, shows highly restricted expression in fat cells [41]. Elevated USP22 expression contributes to reduced treatment efficacy and heightened metastatic potential, ultimately worsening patient survival [42]. USP22 shows elevated expression across multiple tumor types and acts as a cancer-promoting factor by erasing ubiquitin marks from histones, contributing to chromatin structure changes. This leads to the enhancement or preservation of key transcriptional regulators that drive malignancy. Additionally, USP22 targets Cyclin D1, Cyclin B1, and SIRT1 for deubiquitination, thereby modulating cellular division and programmed cell death. USP22 has been characterized as a tumorigenic regulator involved in the progression of malignant transformation. Elevated levels of USP22 have been observed across multiple cancer types, including hepatocellular carcinoma. Notably, its oncogenic activity has been shown to cooperate with HIF1α signaling under specific physiological conditions [40]. It is noteworthy that PPARγ, when elevated by USP22, contributes to enhanced lipid production and tumor growth in connection with Akt2 signaling in hepatocellular carcinoma. Previous findings indicate that USP22 increases PPARγ levels regardless of serum presence, implying that its regulatory effect may occur independently of AKT phosphorylation. This suggests USP22 could function as an upstream modulator within multiple signaling pathways. Given that AKT signaling is notably upregulated in HCC, these data collectively imply that USP22-mediated lipid accumulation may be mechanistically linked to AKT pathway activity [40].

### 2.7. USP39

USP39 has also been widely recognized as a key factor in the regulation of RNA splicing [8]. High levels of USP39 promote uninterrupted splicing activity of specific pre-mRNAs across various types of cancer [43]. Although it was once thought to lack deubiquitinating function due to the absence of catalytic residues such as cysteine, histidine, and aspartate, later research confirmed that USP39 can influence the stability of the DNA damage-associated protein CHK2 via deubiquitination, thereby modulating cellular mechanisms in lung cancer settings [8]. Previous studies have revealed that USP39 facilitates hepatocellular carcinoma development by preserving β-catenin, a key effector of the Wnt/β-catenin signaling cascade, through direct removal of ubiquitin [44]. In the absence of Wnt signals, β-catenin tends to accumulate at cell–cell junctions on the membrane. Within the cytoplasm, it binds to APC and AXIN1, facilitating phosphorylation at its N-terminus. This modification facilitates the recognition of β-catenin by E3 ubiquitin ligases, ultimately targeting it for proteasomal degradation, thereby contributing to the regulation of Wnt signaling in tumor cells [45]. In parallel, USP39 interferes with the splicing and maturation of TRIM26 pre-mRNA, consequently reducing TRIM26-mediated ubiquitination of β-catenin and indirectly promoting tumor progression (Table 1) [44]. TRIM26, also referred to as RNF95 or ZNF173, is an E3 ubiquitin ligase that contributes to immune defense and metabolic disorder regulation by modifying substrate-specific ubiquitination. Earlier investigations demonstrated that TRIM26 acts as a potent inhibitory factor, impeding cell growth, motility, metastasis, and glucose metabolism in hepatocellular carcinoma and papillary thyroid cancer through modulation of the PI3K/AKT pathway or ZEB1 ubiquitination. Additionally, it has been found that TRIM26 hinders hepatic stellate cell activation by promoting the ubiquitination of SLC7A11, thereby reducing the advancement of liver fibrosis via induction of ferroptosis [46].

### 2.8. UCHL3

In hepatocellular carcinoma (HCC), the regulatory interaction between UCHL3 and vimentin has been previously investigated. EEF1A1 is highly upregulated in HCC cells and contributes to malignant traits such as dysregulated cell cycle progression, enhanced invasiveness, metastatic potential, and uncontrolled proliferation. Through mass spectrometry analysis of immunoprecipitated complexes using an anti-UCHL3 antibody, a previous study identified EEF1A1 as a protein that interacts directly with UCHL3 (Table 1) [47]. Vimentin is a representative marker of mesenchymal lineage and has been implicated in promoting tumor expansion, tissue invasion, and poor clinical outcomes in tumor cell. Its elevated expression during epithelial-to-mesenchymal transition (EMT) in tumor cells suggests that inhibiting vimentin could serve as an effective approach to suppress metastatic progression [18]. Beyond its traditional role in aminoacyl-tRNA transport, EEF1A1 has been implicated in modulating signal cascades via direct protein–protein interactions and influencing apoptotic mechanisms by regulating the biosynthesis of death-associated factors. In HCC, EEF1A1 facilitates cellular proliferation through its association with phosphorylated Akt and STAT1, respectively [48]. Hyperactivation of EEF1A1 in hepatocellular carcinoma attenuates the efficacy of Lenvatinib and augments cancer stem-like characteristics. In addition, UCHL3 and EEF1A1 promote the expansion of xenograft tumors in immunodeficient mouse models. These findings suggest that the UCHL3–EEF1A1 signaling axis drives tumor proliferation in vivo [47]. Currently, there are no FDA-approved inhibitors that selectively target UCHL1. TCID has been described as an early-stage inhibitor of UCHL3. However, its concurrent inhibitory activity against UCHL1 limits its specificity and may increase the risk of off-target effects [49].

**Table 1:** DUBs in the regulation process of liver diseases.

Diseases Type	Target DUB	Substrate	Evidence in HCC/Liver Disease Models	Known Inhibitors	Suggested Biomarkers	References
HCC	USP1	SIX1	Suppresses SIX1/Cyclin D1 signaling and sensitizes HCC cells to therapy	GW7647, ML323, KSQ-4279, ISM3091	SIX1, Cyclin D1	[19]
	USP7	STAT3	Reduces PD-L1 and TRIM27/STAT3 signaling	OAT-4828, P5091, FT671	PD-L1, p-STAT3	[25]
	USP11	NF90	Suppresses EMT and metastasis in experimental models	No specific inhibitor	p-AKT	[5]
	USP14	GPX4/HK2	Reduces radioresistance and lenvatinib resistance	IU1, IU1-47	GPX4, GLUT1, CIB1	[35]
	USP19	SOAT1	USP19 removes ubiquitin from SOAT1 at K33 and K48 residues, enhancing its protein stability.	No specific inhibitor	SOAT1	[38]
	USP22	PPARγ	Regulates PPARγ/AKT2-mediated lipogenesis	No specific inhibitor	PPARγ, p-AKT2	[40]
	USP39	β-catenin	β-catenin stabilization and TRIM26 regulation	No specific inhibitor	β-catenin	[44]
	UCHL3	EEF1A1	UCHL3 promotes HCC progression by regulating stemness-associated pathways	No specific inhibitor	EMT-associated proteins	[47]
	OTUB1	TGF-β/FOXM1	Regulates TGF-β and FOXM1 signaling	No specific inhibitor	FOXM1, TGF-β	[50]
Non-alcoholic fatty liver disease	RPN11	METTL3	RPN11–METTL3–ACSS3 axis promotes hepatic lipid accumulation	Capzimin	METTL3, ACSS3	[51]
Liver fibrosis	UCHL1	HIF1α	UCHL1 stabilizes HIF1α and promotes fibrogenesis	LDN-57444	HIF1α, α-SMA, collagen I	[52]
Non-alcoholic steatohepatitis	USP13	IRHOM2	USP13 maintains IRHOM2 stability and regulates inflammation/lipid metabolism	Spautin-1 (USP10/USP13 inhibitor)	IRHOM2, TNF-α	[53]

### 2.9. OTUB1

The human OTU (ovarian tumor) family comprises 16 active deubiquitinating enzymes that are further classified into four distinct subfamilies. Among them, the Otubain subfamily consists of OTUB1 and OTUB2 [54]. OTUB1 has been reported to be highly expressed in HCC and is characterized by the presence of an N-terminal extension and two ubiquitin-binding sites, structural features that contribute to its unique deubiquitinating activity and substrate recognition [55]. Recent studies have reported elevated expression of both OTUB1 and OTUB2 in HCC tissues and cell lines. Mechanistically, OTUB1 promotes tumor invasion and metastasis through activation of TGF-β signaling and enhances therapeutic resistance by stabilizing FOXM1. Given the established roles of TGF-β signaling and FOXM1 in tumor progression and immune evasion, OTUB1 has emerged as a potential target for combination therapeutic strategies. In contrast, OTUB2 contributes to HCC progression by activating NF-κB/p65 and AKT/mTOR signaling pathways, thereby promoting tumor cell proliferation and survival. As these pathways are closely linked to immune regulation and treatment responsiveness, OTUB2 may also represent a promising therapeutic target in the context of immunomodulatory approaches [50].

### 2.10. CYLD

The human OTU (ovarian tumor) family comprises 16 active deubiquitinating enzymes.

## 3. DUB Regulators in Other Liver Disease

### 3.1. RPN11 in Non-Alcoholic Fatty Liver Disease (NAFLD)

Non-alcoholic fatty liver disease (NAFLD) has become the leading global contributor to chronic liver conditions, emerging from relative obscurity [56]. Affecting 38.2% of the global population, NAFLD has rapidly emerged as a major contributor to hepatocellular carcinoma, particularly in the Americas. NAFLD is closely linked to metabolic abnormalities such as insulin resistance, elevated blood glucose, and dyslipidemia [57]. While simple steatosis is typically seen as non-threatening, its progression to liver fibrosis poses a risk for cirrhosis and hepatocellular carcinoma [56]. Rpn11, also known as POH1 in humans, is a conserved metalloprotease component of the proteasome that links ubiquitin removal with substrate degradation [58]. Mice lacking RPN11 specifically in hepatocytes are resistant to diet-induced hepatic fat accumulation, insulin resistance, and inflammatory liver injury. At the molecular level, RPN11 removes ubiquitin from METTL3, stabilizing it and boosting both m6A RNA modification and the expression of the enzyme ACSS3 (Table 1). METTL3, known as Methyltransferase-like 3, operates in mammalian cells by forming a protein complex that catalyzes m6A methylation. This RNA modification participates in various biological activities such as stem cell maintenance, lineage commitment, and DNA lesion repair [51]. ACSS3, in turn, produces propionyl-CoA, which enhances the transcription of lipid metabolism genes through histone propionylation. The RPN11–METTL3–ACSS3–histone propionylation axis is upregulated in the livers of individuals with NAFLD, and increased RPN11 activity correlates with greater hepatic lipid accumulation and disease progression [57].

### 3.2. UCHL1 in Liver Fibrosis

Liver fibrosis poses a serious global health challenge and is marked by abnormal buildup of extracellular matrix (ECM) and disruption of liver architecture, ultimately progressing to advanced liver diseases like cirrhosis and hepatocellular carcinoma. Various factors contribute to the development of liver fibrosis, including chronic viral infections, alcohol-related liver damage, metabolic-associated fatty liver, and autoimmune conditions [59]. The extent of liver fibrosis is closely associated with hepatic functional capacity and is considered a key determinant in the onset of hepatocellular carcinoma (HCC). As a result, cirrhosis ranks as the eleventh leading cause of death worldwide and stands as the fourth most frequent cause of adult mortality in Central Europe [60]. UCHL1 (ubiquitin carboxy-terminal hydrolase L1) belongs to the UCH subgroup of deubiquitinating enzymes and exhibits tissue- and tumor-specific expression patterns. It is one of the most highly expressed proteins in the brain but is nearly absent in tissues such as nasopharynx, stomach, colon, kidney, and in ovarian cancer cells (Table 1) [12]. Numerous tumor cell studies have identified substrates of UCHL1, such as EGFR, TGF-β, SMAD2, and notably, HIF1α, which is particularly relevant to previous study (Table 1). HIF1 is a widely distributed transcription factor composed of two subunits: an oxygen-regulated alpha component and a constitutively active beta partner, also known as ARNT (aryl hydrocarbon receptor nuclear translocator). Previous findings suggest that activation of HIF signaling and oxygen-sensitive pathways plays a key role in modulating liver fibrosis. Deleting HIF1α specifically in hepatocytes results in marked downregulation of fibrosis-promoting growth factors, including PDGFs and FGFs, thereby reducing ECM buildup and fibrotic characteristics. UCHL1 enhances the expression of genes involved in hypoxia response and fibrogentic or proliferative activity by selectively eliminating degradative ubiquitin chains from HIF1α, thereby sustaining its stability and activity [52]. Currently, no fully selective UCHL1 inhibitors are available, with LDN-57444 being primarily utilized as a research tool compound. Experimental studies using liver fibrosis models have demonstrated that LDN-57444 treatment alleviates fibrosis severity and decreases the expression of fibrotic markers, including collagen and α-SMA. These findings suggest a potential therapeutic role for UCHL1 inhibition in liver fibrosis, although further studies are needed to improve inhibitor selectivity and evaluate clinical applicability [61].

### 3.3. USP13 in Non-Alcoholic Steatohepatitis (NASH)

Non-alcoholic steatohepatitis (NASH) is a liver condition marked by cellular damage and histological inflammation. It tends to occur nearly twice as often in males as in females. Since histological confirmation through biopsy is required for diagnosis, identifying cases is challenging. Major contributors to NASH include obesity and type 2 diabetes, with the latter showing a prevalence rate exceeding 37% [62]. As research indicates, USP13 has been identified as a factor that regulates inactive rhomboid protein 2 (IRHOM2), a target in inflammatory diseases, by facilitating its deubiquitination (Table 1). Furthermore, inhibiting USP13 in liver cells interferes with standard hepatic metabolism, which consequently results in abnormal glucose processing, an accumulation of lipids, and heightened inflammation. Ultimately, a deficiency in USP13 leads to the development of non-alcoholic steatohepatitis [53], regulates the ubiquitination status of STING and participates in the antiviral response. It also interacts with FRMD8, influencing various aspects of cellular physiology. Under conditions of metabolic disturbance, IRHOM2 shows pronounced accumulation within proteasomal complexes, highlighting the ubiquitin–proteasome system as a central pathway in controlling IRHOM2 protein levels (Table 1) [63]. IRHOM2 also regulates the maturation and activity of ADAM17, thereby contributing to ectodomain shedding. Consequently, the expression of TNF-α is regulated, ultimately making the expression of TNF-α controlled by IRHOM2 [64].

## 4. Current State of the Field in DUB Targeting

### 4.1. Current Therapeutic Landscape of HCC

Patients with HCC were initially treated with sorafenib as a first-line therapy. However, over the past decade, the development of immune checkpoint inhibitors (ICIs) has led to significant improvements in patient survival. In particular, the combination of atezolizumab and bevacizumab, as well as the combination of durvalumab and tremelimumab, achieved longer survival outcomes compared with previous treatment approaches. Furthermore, pembrolizumab monotherapy and the combination of nivolumab and ipilimumab demonstrated clinical efficacy in the second-line setting and subsequently received FDA approval [65]. The introduction of newer therapeutic strategies has substantially improved the prognosis of patients with HCC. Whereas treatment with sorafenib was associated with a median survival of 10.7 months, the advent of immune checkpoint blockade has contributed to long-term survival outcomes, with a 4-year overall survival rate reaching 25.2%. Current drug development efforts in HCC are primarily directed toward signaling networks involved in immune regulation and angiogenic processes. Major molecular targets under investigation include PD-L1, VEGFR, TGF-β, MAPK, PI3K–AKT, and JAK/STAT pathways [66]. Therefore, this section summarizes current treatment strategies for HCC and highlights the therapeutic potential of DUB-targeted approaches as emerging candidates for future clinical intervention.

### 4.2. Sorafenib (SOR)

Introduced in 2007, sorafenib has remained a cornerstone systemic therapy for HCC. Its development was made possible by advances in molecular cancer research that revealed signaling networks critical for tumor growth and progression. Furthermore, progress in high-throughput compound screening accelerated the drug discovery process, ultimately leading to regulatory approval for advanced renal cell carcinoma after approximately eleven years of development [67]. SOR is a multikinase inhibitor that acts on multiple signaling pathways rather than a single molecular target. By interfering with the Raf/MEK/ERK signaling axis as well as fibroblast growth factor and vascular endothelial growth factor signaling, sorafenib suppresses tumor-associated neovascularization and limits metastatic progression driven by enhanced cellular motility [68]. Despite its clinical benefits, sorafenib is associated with the emergence of therapeutic resistance and several adverse effects in patients with HCC. Mechanisms underlying resistance can be broadly categorized into tumor cell-intrinsic factors and alterations within the tumor microenvironment. In addition, epigenetic dysregulation, ferroptosis-related pathways, epithelial–mesenchymal transition (EMT), and autophagy have been identified as major contributors to the development of sorafenib resistance [69]. The most common adverse effects are diarrhea, hand-foot skin reaction and weight loss with anorexia. Additionally, sorafenib can increase the risk of bleeding, and in rare cases, it can cause myocardial and gastrointestinal perforation [70].

### 4.3. Atezolizumab and Bevacizumab

The combination of atezolizumab and bevacizumab was approved in May 2020 by the FDA as a first-line treatment for patients with advanced hepatocellular carcinoma. The combination therapy led to an increase in overall suvival rate to 67.2% compared with 54.6% in the sorafenib-treated group [71]. Atezolizumab specifically binds to PDL-1, and blocks the interaction between PDL-1 and its receptor, thereby restoring T-cell activation and ultimately blocking the immune evasion. Bevacizumab is an IgGi antibody that binds to VEGF. Because HCC is a highly vascularized tumor, Bevacizumab inhibits VEGF and suppresses HCC’s tumor growth [72]. Atezolizumab and bevacizumab are also associated with adverse effects. The most common adverse events are hypertension and diarrhea. Pneumonitis and thyroid dysfunction are less frequently observed [73]. The introduction of ICI-based immunotherapies has substantially improved clinical outcomes in HCC, particularly when administered in combination regimens that achieve higher response rates and superior survival benefits compared with sorafenib monotherapy. Nevertheless, several limitations remain, including treatment-related toxicities, suboptimal long-term survival outcomes, and the emergence of therapeutic resistance. Previous studies have categorized resistance to immunotherapy into primary (intrinsic) and acquired forms. To address these challenges, current research efforts are focused on developing novel triplet combination strategies alongside established first-line therapies and identifying new molecular targets that may further enhance treatment efficacy and overcome resistance mechanisms [74].

### 4.4. Therapeutic Potential and Challenges of DUB Targeting

Proteins play essential roles in maintaining cellular homeostasis and regulating intracellular signaling pathways. Multiple regulatory mechanisms have evolved to control protein abundance and activity, among which the ubiquitin–proteasome system (UPS) represents one of the major pathways for protein degradation. Because DUBs regulate protein stability by removing ubiquitin chains from substrate proteins, DUB inhibitors have emerged as attractive therapeutic candidates [16]. Unlike immune checkpoint inhibitors (ICIs), which primarily exert indirect antitumor effects through modulation of immune responses, DUB-targeted therapies may directly interfere with oncogenic signaling pathways that drive tumor progression. Furthermore, accumulating evidence suggests that DUB inhibition could provide a potential strategy to overcome therapeutic resistance, which remains a major challenge in the treatment of HCC. Therefore, targeting DUBs may represent a promising complementary approach for improving the efficacy of current HCC therapies. Although no DUB-targeted inhibitor has yet been approved by the FDA, several next-generation compounds targeting USP1, USP7, USP14 and other DUBs have entered clinical development and are being evaluated for their therapeutic potential [75].

### 4.5. USP1 Inhibitor

The development of USP1 inhibitors has been actively pursued for more than a decade. Early examples include pimozide and GW7647, which were reported in 2011 as non-competitive and reversible inhibitors of the USP1/UAF1 complex. Both compounds inhibit USP1 activity by binding outside the catalytic active site. Subsequently, additional USP1 inhibitors, including ML323 in 2012 and SNS-032 in 2022, were identified. These compounds suppress USP1 activity, resulting in increased ubiquitination of substrate proteins such as SIX1 and ultimately inhibiting cancer cell proliferation and tumor growth [75]. Despite these advances, most USP1 inhibitors remain in the preclinical stage. Furthermore, compounds such as ML323 are primarily used as research tools because of limitations related to pharmacokinetic properties, solubility, and stability, which hinder their clinical translation [76]. Nevertheless, recent advances have led to the emergence of USP1 inhibitors that have progressed into clinical development. Among these, KSQ-4279 and ISM3091 represent notable examples. In particular, ISM3091 received regulatory clearance from both the U.S. Food and Drug Administration (FDA) and the National Medical Products Administration (NMPA) in 2023 to enter Phase I clinical trials for the treatment of solid tumors. These developments highlight the growing potential of DUB inhibition as a novel anticancer therapeutic strategy [77]. Additionally, combination treatment with USP1 inhibitors and conventional therapeutic agents for HCC, such as lenvatinib or oxaliplatin, has been reported to suppress therapeutic resistance and further enhance antitumor efficacy in cancer cells [78]. Collectively, these findings suggest that the development of clinically applicable USP1 inhibitors may represent a promising therapeutic strategy for overcoming treatment resistance and improving therapeutic responses in HCC.

### 4.6. USP14 Inhibitor

USP14 is highly expressed in a variety of malignancies, and numerous studies have reported that its elevated expression is associated with poor patient prognosis. Similar observations have been reported in HCC. Mechanistically, USP14 promotes tumor progression through the stabilization of key oncogenic proteins, including HIF-1α and PI3K, both of which are critically involved in tumor growth and survival. Consequently, USP14-mediated signaling may contribute to malignant phenotypes such as cancer cell invasion and metastasis, thereby accelerating disease progression [79]. Emerging evidence suggests that USP14 expression is substantially elevated in HCC cells and tumors exhibiting resistance to lenvatinib, one of the current first-line treatments for HCC. Reduction in USP14 levels was shown to enhance the responsiveness of resistant cells to lenvatinib, supporting its role in drug resistance. At the molecular level, USP14 preserves the stability of calcium- and integrin-binding protein 1 (CIB1) through deubiquitination, thereby protecting it from proteasome-mediated turnover. Sustained CIB1 expression subsequently promotes activation of the PAK1 and ERK1/2 signaling pathways. Moreover, enforced expression of CIB1 was sufficient to confer resistance to lenvatinib, indicating that the USP14–CIB1 signaling axis contributes to the development of therapeutic resistance in HCC [80]. Additionally, in HCC, USP14 has been reported to stabilize glucose transporter 1 (GLUT1), a key regulator of cellular glucose metabolism. Increased GLUT1 expression promotes metabolic reprogramming and contributes to the establishment of an immunosuppressive tumor microenvironment, thereby reducing T-cell activation and facilitating immune evasion. These findings suggest that inhibition of USP14 may enhance antitumor immune responses by restoring T-cell activity and could potentially improve sensitivity to immune checkpoint inhibitors (ICIs) while overcoming therapeutic resistance [78]. Collectively, these findings suggest that the development of USP14 inhibitors holds considerable promise as a therapeutic strategy for the treatment of malignant tumors. Among the currently available compounds, the IU series of USP14 inhibitors has attracted particular attention [81]. Representative examples include IU1 and its derivative IU1-47. IU1 has been reported to induce cell cycle arrest and promote apoptosis in several malignancies, including gastric cancer, cervical cancer, and HCC. Furthermore, IU1-47 was developed as an optimized derivative with enhanced potency and improved pharmacological activity. Recent studies have demonstrated that IU1-47 effectively suppresses tumor-promoting signaling pathways and may attenuate immune evasion mechanisms, highlighting its potential as a next-generation USP14-targeted therapeutic agent [82].

## 5. Perspective

This review focuses on the potential roles of deubiquitinating enzymes (DUBs) in hepatocellular carcinoma (HCC), a malignancy characterized by both high incidence and mortality, as well as in related liver diseases. It highlights DUBs as promising therapeutic candidates to overcome the limitations of conventional chemotherapy, including adverse effects, and the restricted applicability of surgical interventions such as resection and transplantation. In addition, the review introduces various classes of DUBs alongside E3 ubiquitin ligases, emphasizing their regulatory roles and therapeutic relevance. To date, over one hundred deubiquitinating enzymes have been identified, and these enzymes function as key modulators of the ubiquitin–proteasome system. They are categorized into six major groups, including ubiquitin-specific proteases (USPs), JAB1/MPN/MOV34 metalloproteases (JAMMs), ubiquitin C-terminal hydrolases (UCHs), ovarian tumor proteases (OTUs), motif interacting with ubiquitin-containing novel DUBs (MINDYs), and Josephin family members. Given their regulatory roles, DUB modulation may provide valuable insights into the molecular basis of hepatocellular carcinoma and to improve therapeutic responsiveness in HCC and related liver diseases [83].

## 6. Conclusions

Despite continuous advances in science and medical technology, cancer remains a major global health burden. Among various malignancies, hepatocellular carcinoma (HCC) is considered one of the most lethal cancers owing to its heterogeneous etiology, difficulty in early diagnosis, high metastatic potential, and poor prognosis. HCC accounts for approximately 90% of all primary liver cancers and frequently develops in the setting of chronic liver diseases such as chronic hepatitis and cirrhosis. Current therapeutic strategies for HCC include surgical interventions and systemic therapies. Over the past decade, systemic treatment for HCC has evolved from tyrosine kinase inhibitors (TKIs), such as sorafenib and lenvatinib, to immune checkpoint inhibitor (ICI)-based combination regimens, including atezolizumab plus bevacizumab and durvalumab plus tremelimumab. Although these advances have improved clinical outcomes, several challenges remain, including limited response rates, intrinsic and acquired resistance, and treatment-related adverse effects. Recent studies have highlighted the critical roles of deubiquitinating enzymes (DUBs) in regulating tumor progression, therapeutic resistance, immune evasion, and metabolic processes in HCC. Furthermore, accumulating evidence suggests that pharmacological inhibition of oncogenic DUBs may enhance the efficacy of existing therapies and help overcome resistance mechanisms. Several next-generation inhibitors targeting DUBs, including USP1 and USP14, have entered clinical development, supporting the translational potential of DUB-targeted therapeutic strategies. Therefore, the development and clinical validation of selective DUB inhibitor modulation may represent important future directions in HCC research. Moreover, combination strategies incorporating DUB-targeted agents with existing targeted therapies and immune checkpoint inhibitors may provide a novel therapeutic approach for improving treatment responses and overcoming therapeutic resistance in patients with HCC.

## Data Availability

No new data were created or analyzed in this study. Data sharing is not applicable to this article.
